# Long‐term in vitro persistence of magnetic properties after magnetic bead‐based cell separation of T cells

**DOI:** 10.1111/sji.12924

**Published:** 2020-07-17

**Authors:** Aicha Laghmouchi, Conny Hoogstraten, J. H. Frederik Falkenburg, Inge Jedema

**Affiliations:** ^1^ Department of Hematology Leiden University Medical Centre Leiden The Netherlands

**Keywords:** MACS isolation, magnetic nanoparticles, T cells

## Abstract

Magnetic‐activated cell sorting (MACS) using magnetic nanoparticles coated with specific antibodies is commonly used in immunology research. For in vitro isolation purposes, it is important to know to what extent the magnetic properties remain present in the isolated cell populations and whether it has consequences for sequential isolations. We hypothesized that only upon cell division, cells will lose their magnetic properties via dilution of the particles in/on their daughter cells. We analysed residual magnetic properties of cells that divided vs cells that did not divide after magnetic bead‐based cell separation. As a model, we isolated T cells using beads targeting the non‐modulating surface molecule CD45RO. Cells were labelled with the cell division tracking dye PKH and cultured under different conditions to induce variable degrees of cell division. We demonstrate that T cells that underwent no, or only minimal, cell divisions after MACS retained magnetic properties for up to at least 2 weeks of in vitro culture. The presence of nanoparticles was detected on their cell surface and intracellularly using Labeling Check reagent. These results have important consequences for procedures requiring repetitive isolation rounds after in vitro culture.

## 1 INTRODUCTION

Methods to enrich for certain cell populations are commonly used in the field of immunology, besides fluorescence‐activated cell sorting (FACS), magnetic bead‐based cell separation can be applied to isolate cell populations of interest from peripheral blood, bone marrow, other tissues or cell cultures for in vivo administration or for in vitro applications. Magnetic cell separation techniques are available from different manufacturers using different magnetic particles and isolation procedures.^1^, ^2^, ^3^, ^4^, ^5^, ^6^, ^7^, ^8^, ^9^

A commonly used magnetic cell separation method is the MACS® system using nanosized superparamagnetic particles (nanobeads 20‐100 nm; Miltenyi Biotec), separation columns and a magnet.^2^, ^3^, ^4^ Cells labelled with MACS beads are captured by the magnetic field of the separator, whereas unlabelled cells pass the magnetic field and end up in the flow‐through fraction. Cells can be either directly labelled with magnetic nanoparticles coated with antibodies specific for a surface marker, or indirectly labelled using primary antibodies conjugated with a fluorochrome or biotin and magnetic beads coated with fluorochrome‐ or biotin‐specific antibodies. Since the beads are composed of iron oxide and polysaccharide, it is advertised by the manufacturer that these magnetic nanoparticles are biodegradable, which implies a loss of magnetic properties over time in the MACS isolated cell populations.^1^, ^2^, ^3^, ^4^ Other studies have investigated the localization and clearance of MACS beads and found that the particles were present both on the extracellular and intracellular sides of the cells. These studies describe the internalization process in metabolic active cells which leads to the degradation of the beads within the lysosomes.^10^, ^11^

It has been shown that the MACS method can be used for efficient enrichment of cell populations that can be safely directly administered to patients with retained functionality (eg CD34‐positive hematopoietic progenitor stem cells).^12^, ^13^ However, for in vitro studies it is also important to know if and for how long the magnetic properties remain present in the isolated cell populations and whether it is possible to perform sequential MACS isolations. The aim of this study is to analyse the persistence of magnetic nanoparticles after a culture period of 2 weeks to allow subsequent isolation of antigen‐specific T cells based on activation marker expression after restimulation. We hypothesize that upon cell division cells will rapidly lose magnetic properties via dilution of the nanoparticles on the daughter cells.

To study this hypothesis, we used CD45RO beads to target the isoform of CD45 (prototypic receptor‐like protein tyrosine phosphatase, an essential regulator of signal transduction pathways in immune cells) to separate memory T cells from pre‐enriched T‐cell populations. The use of CD45RO magnetic nanoparticles functions as an illustrative model for MACS isolations targeting a stable and non‐modulating cell surface marker to be able to track the magnetic nanoparticles on the surface of cells.^14^ Moreover, memory T cells gave us the opportunity to perform in vitro stimulation experiments to induce different levels of cell division to test our hypothesis.

We demonstrate that in the absence of post‐isolation proliferation, CD45RO^pos^ (positive) cells retain magnetic properties up to at least 2 weeks after the primary magnetic bead‐based isolation, whereas upon vigorous post‐isolation proliferation, CD45RO^pos^ cells lose magnetic properties. These results have important consequences for procedures requiring repetitive isolation rounds after in vitro culture and illustrate that careful selection of an appropriate cell enrichment strategy is important when isolating cells for further in vitro application.

## 2 MATERIALS AND METHODS

### 2.1 Positive selection and untouched isolation of memory T cells

Mononuclear cells (PBMC) were isolated by Ficoll‐Isopaque separation from peripheral blood that was obtained from healthy donors after informed consent. Initial enrichment of T cells was performed by negatively selected (untouched) MACS isolation using the Pan T cell isolation kit (130‐096‐535; Miltenyi Biotec). In the same incubation step extra CD14 beads (CliniMACS CD14 Reagent, 200‐070‐118; Miltenyi Biotec) were added for more stringent monocyte depletion. Both the Pan T cell isolation kit and the CD14 beads were used according to manufacturer's instructions. Memory T cells were subsequently enriched via a direct isolation method by positive selection using CD45RO beads (130‐046‐001; Miltenyi Biotec), or as a non‐magnetically labelled control via untouched isolation procedure by depletion of naïve and effector T cells using CD45RA beads (130‐045‐901; Miltenyi Biotec). These isolations were done by immunomagnetic separation using LS columns (130‐042‐401, Miltenyi Biotec) and the midi‐MACS system (Miltenyi Biotec) according to manufacturer's instructions (schematic overview of the isolations is shown in Figure S1A). Isolation efficiencies were analysed by incubating cells with fluorescein isothiocyanate (FITC)‐labelled CD45RO (MHCD45RO01, Clone UCHL1; Invitrogen), peridinin chlorophyll protein (PerCP)‐labelled CD3 (345766, Clone SK7; BD Biosciences) and allophycocyanin (APC)‐labelled CD45RA (550855, Clone HI100; BD Pharmingen) monoclonal antibodies for 30 minutes at 4°C. Fluorescent events were analysed using a calibrated FACSCalibur (BD Biosciences), Cellquest software (BD Biosciences) and FlowJo software (FlowJo LLC). The gating procedure was performed after applying fitting instrument settings and compensation. The gating strategy of the different cell fractions started with the gating of lymphocytes using the forward and sideward scatter followed by plotting the cells for CD45RO and CD45RA expression.

### 2.2 Induction of proliferation by in vitro stimulation

To track the extent of cell division, isolated memory T cells (by either positive selection or untouched isolation) were labelled with the PKH26 red fluorescent cell linker kit for general cell membrane labelling according to manufacturer's instructions (PKH26GL; Sigma‐Aldrich). The medium used for cell culture was Iscove's modified Dulbecco's medium (IMDM; 12‐722F; Lonza) supplemented with 10% pooled human serum, 100 U/mL penicillin/streptomycin (17‐602E; Lonza) and 3 mmol/L l‐glutamine (17‐605E; Lonza). To assess the residual magnetic properties of cells that underwent no or multiple cell divisions after positive selection, variable levels of proliferation were induced using three different stimulation conditions. The first condition consisted of culture in medium supplemented with a minimal amount of cytokines (1 ng/mL IL‐7 (130‐095‐367; Miltenyi Biotec) and 0.01 ng/mL IL‐15 (130‐095‐760; Miltenyi Biotec)) allowing cell survival, but no overt induction of proliferation. The second condition comprised induction of (partial) proliferation of only the allo‐reactive T‐cell subset upon stimulation with complete HLA‐mismatched, 50 Gray‐irradiated EBV‐LCL (50:1, T cells to EBV‐LCL ratio; EBV‐LCL were generated using standard procedures^15^) in the same medium. The third condition consisted of vigorous, polyclonal, antigen‐independent induction of proliferation using medium supplemented with 800 ng/mL phytohemagglutinin (PHA‐HA16; R30852801; Thermo Fisher Scientific) and 100 IU/mL IL‐2 (751134; Novartis). The schematic overview of this procedure is described in Figure S1B. The analyses of cell division were performed using a FACSCalibur, Cellquest software and FlowJo software after applying suitable instrument settings and compensation. Lymphocytes were gated based on the forward and sideward scatter and were then plotted for CD3 against PKH26. The quantification of the experiment was performed in Prism 8 with the *t* test as statistical analysis method.

### 2.3 Analysis of residual magnetic properties in time after initial magnetic bead‐based cell separation

The residual magnetic properties of non‐divided (PKH^bright^) and divided (PKH^dim^) memory T cells were analysed at 2 weeks after initial magnetic bead‐based cell separation and subsequent in vitro culture (schematic overview is described in Figure S1C). Cells were counted using Eosin Y (E6003‐25G; Sigma‐Aldrich) and loaded onto MACS columns without additional magnetic labelling; both the column‐retained and flow‐through fractions were collected and counted. To analyse residual presence of both the monoclonal antibodies by which the magnetic nanoparticles bind to the cells and the magnetic nanoparticles on the cell surface, cells were incubated with respectively goat‐anti mouse‐Ig antibodies conjugated with FITC (349031; BD Biosciences) and specific labelling of the dextran coating of microbeads by using Labeling Check Reagent‐APC (130‐122‐228; Miltenyi Biotec) or Labeling Check Reagent‐PE (130‐095‐228; Miltenyi Biotec) for 30 minutes at 4°C. The presence of magnetic nanoparticles was also analysed intracellularly, by harvesting cells and performing initial cell surface staining with Labeling Check Reagent‐APC for 30 minutes at 4°C. Cells were then washed in PBS and fixed with 1% paraformaldehyde for 8 minutes at 4°C. For permeabilization, cells were washed in PBS with 0.1% saponin (S7900‐100G; Sigma‐Aldrich) and incubated for 30 minutes at 4°C. Then, cells were stained with or without Labeling Check Reagent‐APC for 30 minutes at 4°C, washed and analysed using a FACSCalibur, Cellquest software and FlowJo software. The gating procedure was performed after applying fitting instrument settings and compensation. The presence of magnetic nanoparticles was analysed by the staining with Labeling Check reagent. Lymphocytes were initially gated based on the forward and sideward scatter followed by the selection of CD3^+^ cells. The Labeling Check staining was then plotted to distinguish the Labeling Check negative and positive populations for further analyses like the tracking of cell division in both populations. The quantification of the experiment was performed in Prism 8 with the *t* test as statistical analysis method.

### 2.4 Subsequent isolation of allo‐reactive T cells based on the expression of the activation marker CD137

To assess whether residual magnetic properties of cells that did not undergo multiple cell divisions upon initial positive selection hampers sequential isolation procedures, positively selected or untouched (non‐magnetically labelled control) isolated memory T cells were stimulated with completely HLA‐mismatched, 50 Gray‐irradiated EBV‐LCL (50:1 T cells: EBV‐LCL ratio) in IMDM, supplemented with 10% pooled human serum, 100 U/mL penicillin/streptomycin (Lonza) and 3 mmol/L l‐glutamine (Lonza) to induce an allo‐reactive T‐cell response. At 2 weeks after initial stimulation, cultures were restimulated with HLA‐mismatched EBV‐LCL at a 10:1 ratio. Allo‐reactive T cells were isolated 24 hours after restimulation by staining for the activation marker CD137 with CD137‐APC (550890, Clone 4B4‐1, BD) for 30 minutes at 4°C and labelling with anti‐APC microbeads (130‐090‐855; Miltenyi Biotec) followed by magnetic bead‐based cell separation using MACS LS columns and a midi‐MACS cell separator, according to the manufacturer's instructions (Miltenyi Biotec). The schematic overview of this procedure is described in Figure S1D. To analyse the purity of the CD137 isolations, the expression of CD137 on the cells in the different fractions was analysed by first gating on the lymphocytes using forward and sideward scatter followed by the plotting of CD3 against CD137 and Labeling Check reagent. Fluorescent events were analysed using a FACSCalibur, Cellquest software and FlowJo software after applying fitting instrument settings and compensation. The quantification of the experiment was performed in Prism 8 with the t test as statistical analysis method.

### 2.5 Ethics approval statement

Donors had given written informed consent to the storage of biomaterials in the LUMC Biobank, and the use of these materials was approved by the institutional medical ethical committee (protocol number B 16.039).

## 3 RESULTS

### 3.1 Majority of positively selected T cells undergoing no or limited proliferation retain magnetic properties even after 2 weeks of culture

**FIGURE 1:**
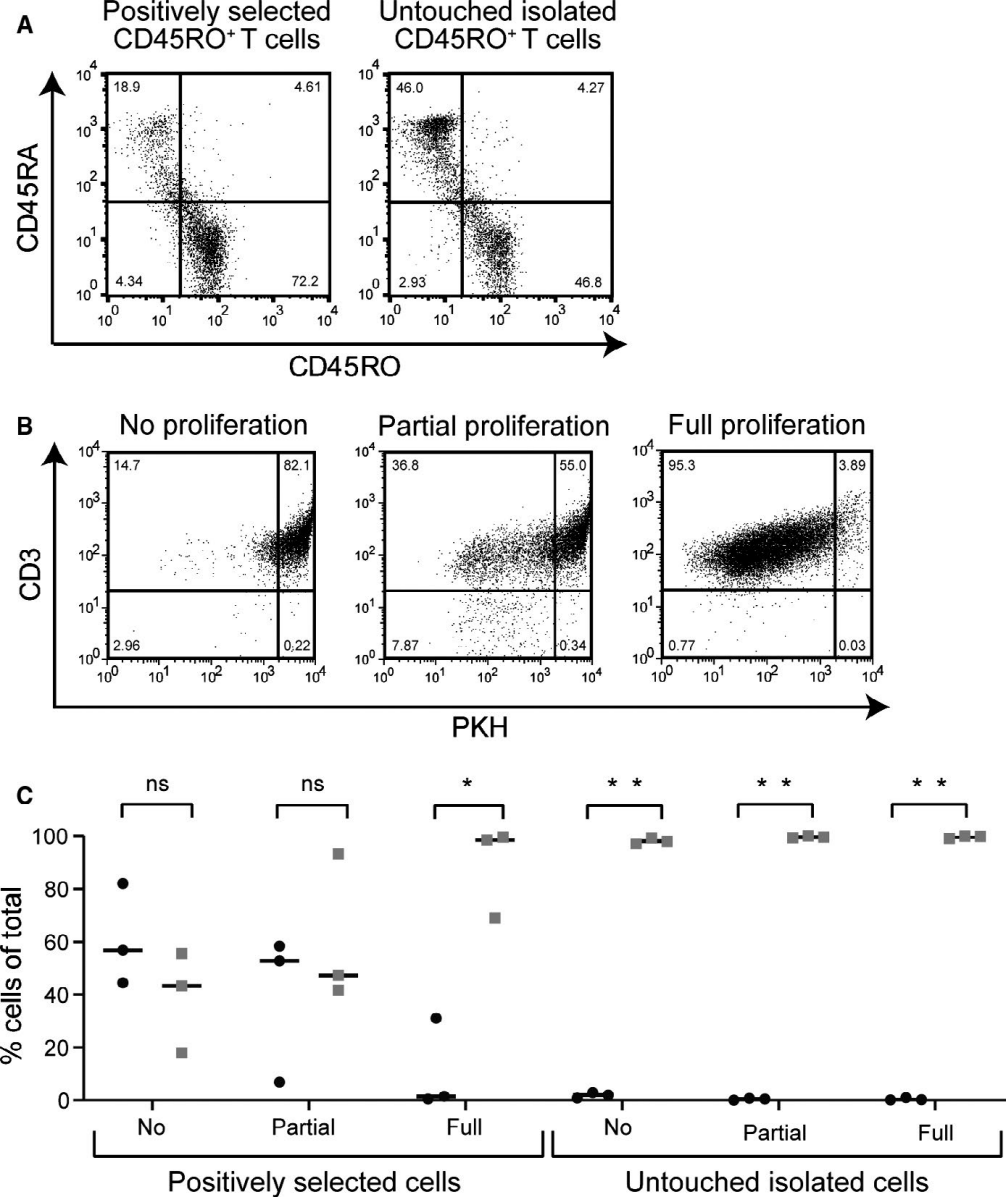
Positively selected memory T cells undergoing no or limited proliferation retain magnetic properties. A, Dot plots of representative (from nine experiments in total) viable positively selected (CD45RO^pos^) memory T cells and control, viable untouched isolated (CD45RA^neg^) memory T cells at day 0. The gating was initially performed on lymphocytes using forward and sideward scatter, and then, cells were plotted for CD45RO and CD45RA. B, Positively selected memory T cells were cultured for 2 wk under different stimulation conditions (representative dotplots (from n = 3) of viable cells are shown, starting from left: without stimulation (cytokines only) resulting in no/minimal PKH dilution (no/minimal proliferation (cytokines only)); partial stimulation inducing proliferation of a portion of T cells (allo‐reactive T cell response), a‐specific stimulation inducing proliferation of almost all cells (PHA stimulation)). The gating on the lymphocytes was done using forward and sideward scatter, followed by plotting for CD3 and PKH to track the cell division. C, Both positively selected and untouched isolated memory T cells were applied after the 2 wk of in vitro culture onto MACS columns without additional magnetic bead labeling. Quantification was performed on viable cells, the cell frequencies in the flow‐through fractions (grey squares) and the column‐retained fractions (black circles) are presented as percentages of total viable cells applied onto the separation columns. The experiment was repeated three times using three different donors. The statistical analysis was performed with t test in Prism 8 (*P*‐value: *<.001 and **<.000001)

To investigate the residual magnetic properties of cells that underwent multiple cell divisions vs cells that did not or only minimally divide after positive selection targeting a non‐modulating surface antigen, memory T cells isolated based on expression of CD45RO were used as a model system. Memory T cells were enriched by magnetic‐activated cell sorting (MACS) from purified T cells via direct positive selection using CD45RO microbeads. As a non‐magnetically labelled negative control, memory T cells were selected from purified T cells using an untouched isolation method based on depletion of naïve and effector T cells (example of isolation purities shown in Figure 1A). The isolated memory T‐cell populations were labelled with the proliferation tracking dye PKH, and subsequently, variable levels of memory T‐cell proliferation were induced using different in vitro culture conditions: no/minimal proliferation using cytokines only, proliferation of only the allo‐reactive fraction within the memory T‐cell compartment upon stimulation with HLA‐mismatched EBV‐LCL (partial proliferation), or induction of vigorous, antigen‐independent, polyclonal proliferation upon PHA stimulation (full proliferation, Figure 1B). No difference was observed in the proliferation status of positively selected memory T cells and untouched isolated memory T cells under the different stimulation conditions (Figure S2), indicating that the presence of magnetic particles did not influence the proliferative potential of the cells.

To analyse the residual magnetic properties of cells that underwent multiple cell divisions vs cells that did not or only minimally divide after magnetic bead‐based cell separation, the different memory T‐cell populations from three independent experiments were applied onto magnetic columns after 2 weeks of in vitro culture following the initial isolation procedure without additional magnetic bead labelling. In Figure 1C, the quantification of viable cells that ended up in either the column‐retained (black circles) or the flow‐through fractions (grey squares) illustrates that the frequencies of cells that were retained by the MACS separator system were inversely correlated with the amount of proliferation induced after the initial magnetic bead‐based isolation procedure, resulting in the highest proportion of viable cells retained on the column in the condition where no/minimal proliferation occurred, whereas in the condition where maximal proliferation was induced the majority of cells ended up in the flow‐through fraction. As expected, after expansion of the non‐magnetically labelled, untouched isolated memory T‐cell populations almost all cells ended up in the flow‐through fractions (Figure 1C).

### 3.2 A significant proportion of the positively selected memory T cells that do not undergo multiple cell divisions retain magnetic nanoparticles on their cell surface

**FIGURE 2:**
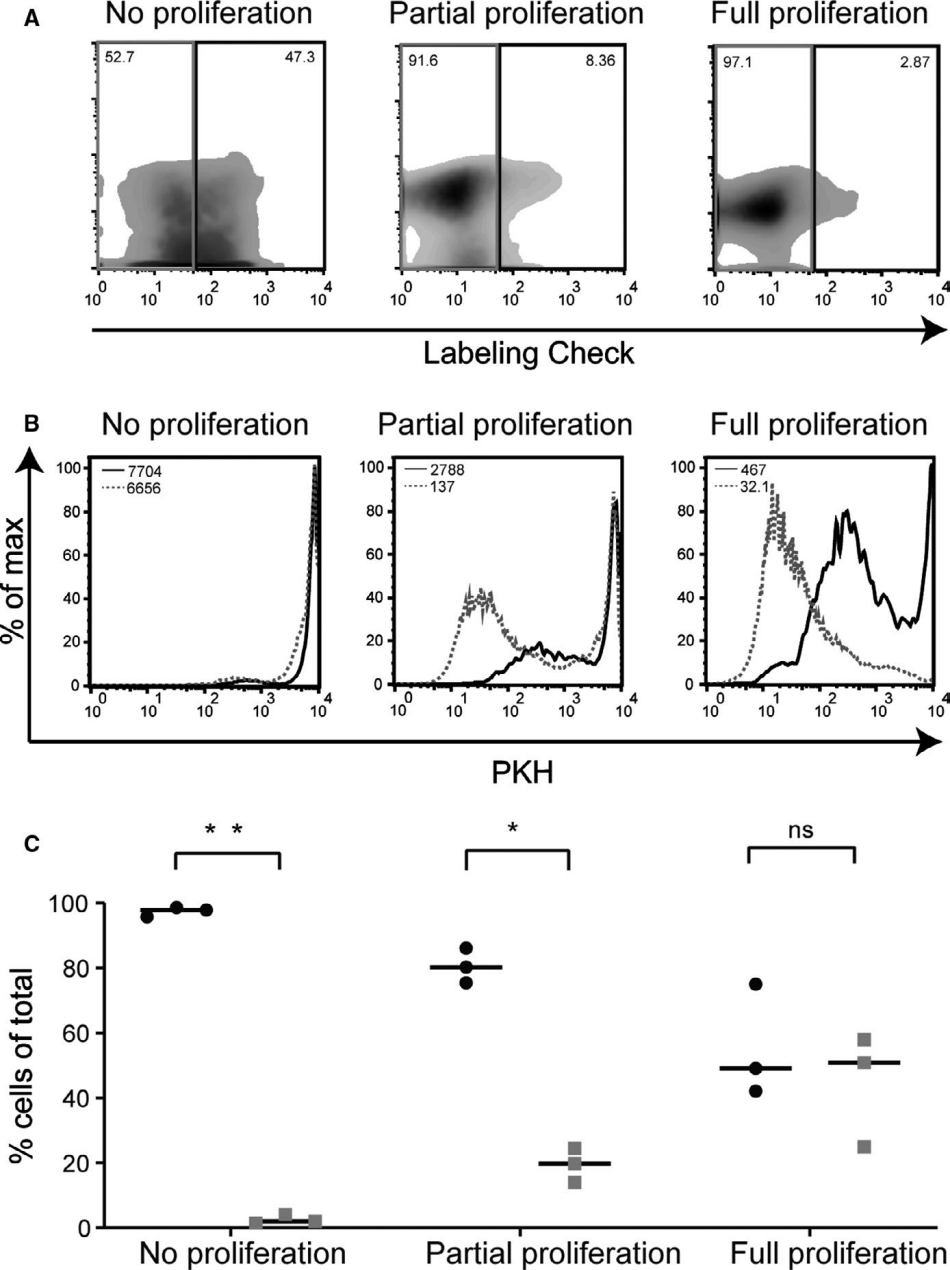
Non‐divided, positively selected memory T cells retain magnetic nanoparticles on their cell surface. A, Representative (n = 3) density plots of viable cells show the Labeling Check reagent staining (positive for staining indicated by the black boxes and negative by the grey boxes) after 2 wk of in vitro culture under different stimulation conditions (from left to right: no/minimal proliferation (cytokines only); proliferation of a portion of T cells (allo‐reactive T cell response), and full, a‐specific proliferation of almost all T cells (PHA stimulation)). The gating of lymphocytes was based on forward and sideward scatter after which the CD3^+^ cells were selected and plotted for Labeling Check staining. The untouched isolated memory T cells were used to set the gates for the Labeling Check. B, From a representative (of n = 3) experiment, the Labeling Check reagent positive cells (black curves) and the Labeling Check reagent negative cells (grey dashed curves) were plotted as histograms for their PKH staining and compared by generating overlays of the two populations. C, After 2 wk of in vitro culture, the cell populations were again applied onto a MACS separator column. Composition quantification was done of the column‐retained fractions of the different stimulated cell populations by calculating the frequencies of dividing cells (grey squares, PKH^dim^) and non‐dividing cells (black circles, PKH^bright^). The column‐retained fractions were analyzed by gating on the lymphocytes using the forward and sideward scatter followed by selection of CD3^+^ cells to be plotted for PKH staining. The experiment was repeated three times using three different donors. The statistical analysis was performed with *t* test in Prism 8 (*P*‐value: *<.0005 and **<.000001)

To study whether the residual magnetic properties of memory T cells that did not undergo multiple cell divisions after magnetic bead‐based positive selection was due to the persistence of magnetic nanoparticles on the cell surface, flow cytometry analysis was performed using Labeling Check reagent that specifically stains the dextran coating of the nanoparticles used for isolation. After 1 week of culture, the magnetic properties were still present on a significant proportion of the cells under the different stimulation conditions (Figure S3). In Figure 2A, the flow cytometry analysis is shown of the positively selected memory T cells after 2 weeks of in vitro culture. Part of the cells stained positive for the Labeling Check reagent (black squares in density plots), indicating residual presence of magnetic nanoparticles on their cell surface. The frequency of Labeling Check positive cells was high in the conditions where no (44.9%‐62.7%) or partial (8.36%‐10.5%) proliferation was induced after the initial magnetic bead‐based separation procedure (Figure 2A). On the Labeling Check positive cells, the presence of monoclonal antibodies (by which the magnetic nanoparticles bind to the cells) was shown using goat‐anti‐mouse‐Ig antibodies (Representative example in Figure S4).

Analysis of the proliferation status (PKH staining) gated on the Labeling Check positive (black curve) and negative (grey dashed curve) populations further illustrated that the Labeling Check positive cells showed a higher PKH intensity, indicating that the nanoparticles were mainly retained on the surface of cells that underwent no, or only minimal cell division after the initial magnetic bead‐based cell separation procedure (Figure 2B, Figure S5). When an additional intracellular labelling was performed with Labeling Check reagent, the intensity of the staining with Labeling Check reagent was 1.7× (median fluorescence intensity (MFI)) higher compared to the staining intensity after cell surface labelling only, indicating intracellular presence of magnetic nanoparticles (Figure S6).

After 2 weeks of in vitro culture, the different cell populations from three independent experiments were applied again onto a MACS separator column without additional magnetic bead labelling. The frequencies of (PKH^bright^) and (PKH^dim^) cells were quantified in the column‐retained fractions after secondary application onto the MACS separator. The quantification of non‐divided (PKH^bright^; black circles) and divided (PKH^dim^; grey squares) viable cells in the column‐retained fractions showed that mainly non‐divided cells were captured by the separation column (Figure 2C), especially in those conditions were sub‐optimal proliferation was induced. In the case of the condition were full proliferation was achieved, the column‐retained fraction is composed of a very low number of a‐specifically bound cells and almost all of these cells ended up in the flow‐through fraction (see also Figure 1C).

### 3.3 Residual magnetic properties of non‐divided cells disturb procedures requiring repetitive isolation rounds after in vitro culture

**FIGURE 3:**
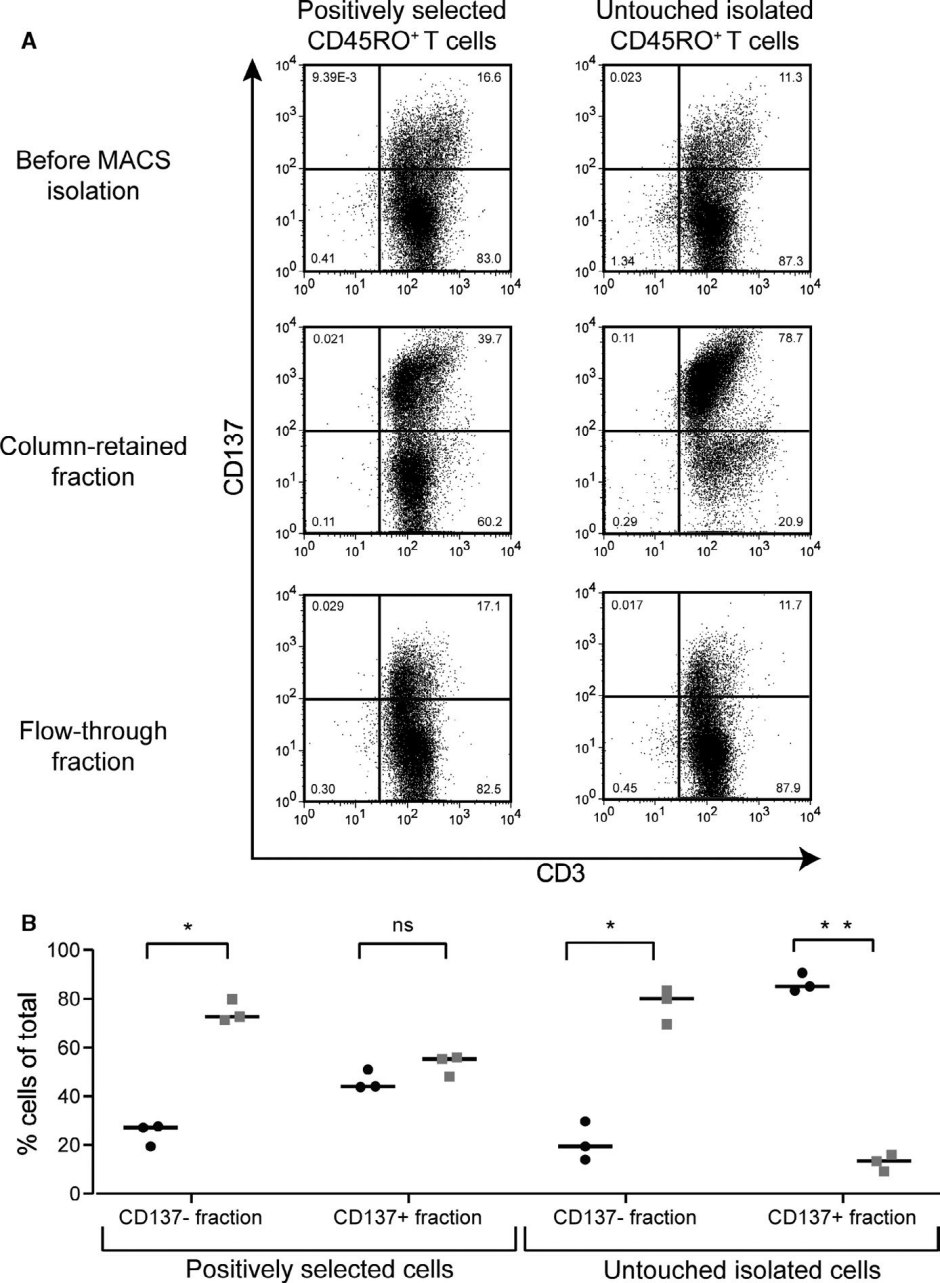
Residual magnetic properties disturb purities in repetitive isolation rounds. A, Representative (of n = 3) dotplots of viable cells are shown for allo‐reactive T cell responses generated from positively selected memory T cells or untouched memory T cells (the quadrants were set based on the unstained samples). The gating of lymphocytes was based on the forward and sideward scatter after which they were plotted for CD3 and CD137 expression. After 2 wk in vitro culture of the two‐differential isolated memory T cell populations, the cells were restimulated with HLA‐mismatched EBV‐LCL. CD137 (activation marker) staining was performed 24 h after the restimulation. To isolate the allo‐reactive (activated) T cells, staining with CD137‐APC and then indirect isolation with anti‐APC magnetic beads was performed. After application on the separation columns, both the column‐retained fractions and the flow‐through fractions were analyzed for CD137 expression to assess the purity of the allo‐reactive (activated/CD137^pos^) T cell isolation. B, Scatter plot with the quantification of three independent experiments shows the percentages of CD137^pos^ cells (black circles) and CD137^neg^ cells (grey squares) of total for both the CD137 negative fraction and the CD137 positive fraction. The experiment was repeated three times using three different donors. The statistical analysis was performed with *t* test in Prism 8 (*P*‐value: *<.001 and **<.00005)

To check whether the residual magnetic properties caused by persistence of magnetic nanoparticles on the cell surface of T cells that did not undergo multiple cell divisions after initial magnetic bead‐based positive selection indeed disturb a sequential selection procedure after 2 weeks of culture, we used allo‐reactive T cell responses induced by stimulation with HLA‐mismatched EBV‐LCL as a model to create a mixture of divided (allo‐reactive) and non‐divided cells. These cultures were restimulated with irradiated HLA‐mismatched EBV‐LCL 2 weeks after initial stimulation. At 24 hours after restimulation, the responding allo‐reactive T cells were labelled with CD137‐APC and anti‐APC‐beads, and applied on the MACS separator. The purities of CD137 positive T cells in the CD137 positive (column‐retained fraction) and the CD137 negative (flow‐through fraction) fractions were determined by flow cytometry with CD137‐specific antibodies and Labeling Check reagent (Figure S7). As shown for a representative example in Figure 3A, significant contamination of CD137 negative cells was found in the column‐retained fractions when this strategy was performed using memory T cells that were obtained by initial positive isolation with CD45RO beads in comparison to the untouched isolated memory T cells. Quantification of three independent experiments shows a similar picture. The CD137 positive fractions were clearly contaminated with 48.0%‐55.9% CD137 negative cells (grey squares) in the case of the positively selected memory T cells, whereas in the case of the untouched isolated memory T cells the contamination was 9.1%‐16.0% (Figure 3B).

These data illustrate that residual magnetic properties of cells that did not undergo multiple cell divisions after initial magnetic bead‐based positive selection disturb the purity in procedures requiring repetitive isolation rounds after in vitro culture. To avoid this problem, an alternative cell enrichment method using reversible magnetic labelling can be applied to isolate cells of interest. For this purpose, CD45RA‐Fab Streptamers were used (methods and results described in Methods S1 and Figure S8). This procedure showed efficient isolation of CD45RA^pos^ cells, and after the D‐biotin dissociation step (removing the magnetic microbeads from the isolated cells), no cells were bound to the magnet (Figure S8) and all cells were retained in the non‐magnetic fraction.

## 4 DISCUSSION

In this study, we demonstrate that after magnetic bead‐based isolation, cells that undergo multiple cell divisions lose magnetic properties by dilution of the MACS beads over the daughter cells. However, cells that do not divide after MACS retain magnetic properties for up to at least 2 weeks. We demonstrate that magnetic nanoparticles are preserved on the cell surface of a large proportion of these cells and were partially internalized during the culture period, together leading to long‐term preservation of the magnetic properties in these cells hampering subsequent isolation strategies.

The MACS method has been shown to be a very efficient magnetic isolation technique for both in vivo and in vitro applications and without indications that the magnetic particles affect functionality of the isolated cells. MACS is extensively used for the enrichment of CD34‐positive hematopoietic progenitor stem cells for adoptive transfer resulting in proper reconstitution and functional hematopoietic cells in vivo without obvious harmful clinical effects of the magnetic particles.^12^, ^13^ Another example is the in vitro isolation of functional virus‐specific and leukaemia‐reactive T cells.^16^, ^17^ Although the functionality of the isolated cells seems not to be affected, we show that the persistence of magnetic properties in isolated cell populations makes it difficult to perform sequential MACS isolations in vitro.

It is unknown whether this phenomenon is similar for other magnetic isolation techniques that use different magnetic particles and isolation procedures, like Dynal® (microbeads with size 1‐3 µm; Invitrogen),^5^ BD with their IMag® particles (100‐500 nm; BD Biosciences), EasySep® magnetic particles (about 150 nm; Stem Cell Technologies),^6^ MagCellect® Cell Selection Kit (nanobeads with size around 150 nm; R&D Systems (Techne)). For these techniques, the cells are bound by microbeads or by nanobeads (100‐500 nm) and can be separated from unwanted cells by placing the tube containing the mix of cells in a magnetic field.^2^, ^5^

The gold standard for magnetic cell separation remains MACS technology, as the efficiency of specific isolation is high and it has been shown in different studies that the magnetic particles have no effect on the functionality of isolated cells.^12^, ^13^, ^16^, ^17^ Miltenyi describes on its website different isolation strategies where multiple sequential isolation steps are used to obtain the cell subset of interest, but they do not describe a strategy with multiple positive selection steps at one time point as the first positive selection would interfere with the second positive selection step. Repetitive isolation steps have also been used in the context of in vitro generation of antigen‐specific T cells; however, in this case the primary isolation was untouched and the second, after 2 weeks of culture, was a positive selection procedure.^16^, ^17^ In our study, we have used the MACS technology for repetitive positive selection steps, with a culture period of 2 weeks in between as the assumption was that the magnetic beads would be gone by then. Our findings suggest that caution must be exercised when using MACS for repetitive positive isolation steps also after prolonged in vitro culture. To avoid isolation problems, the choice for untouched MACS isolations would already help. But positive selection remains the best way to isolate populations with a high purity, and the choice for untouched isolation is not always possible, especially under GMP conditions. Therefore, it would be interesting to use a technique by which the magnetic labelling is immediately reversible to obtain purified unlabelled cells so that the magnetic labelling will not be maintained on the cell surface and also not be taken up by the cells during subsequent in vitro culture procedures.

Miltenyi recently has introduced the REAlease Immunomagnetic Separation Technology that allows for sequential magnetic isolation by positive selection by removal of beads from the cells. Unfortunately, this technology is available for only a limited number of CD markers (eg CD3, CD4, CD8, CD19, CD45, CD56 and CD62L). However, in the case of the REAlease Fluorochrome Technology the list of CD markers that can be labelled is more extensive and includes also CD45RO. Therefore, it will probably not be long before the Immunomagnetic Separation will also be available for other REAlease Fluorochrome Complexes. Another approach is when cell surface molecules are bound by low‐affinity antibody‐derived Fab‐fragments multimerized with the Strep‐Tactin complex (Streptamers).^18^ After magnetic isolation, d‐biotin can be added to the isolated cells to compete with the binding sites on Strep‐Tactin and therefore will result in the dissociation of Streptamers from the positively selected cells.^7^, ^8^, ^9^ So, a variety of cell enrichment procedures are available to select the most appropriate isolation procedure that suits the purpose of the study for which the cells of interest are used.

## CONFLICT OF INTEREST

The authors declare no conflicts of interest.

## AUTHOR CONTRIBUTIONS

AL, CH, JHFF and IJ designed and analysed the experiments. AL and CH performed the experiments. AL, JHFF and IJ wrote the manuscript.

## Supporting information

Appendix S1
