# Supplementary material for: Long‐term in vitro persistence of magnetic properties after magnetic bead‐based cell separation of T cells
Source: Scand J Immunol. 2020 Jul 17;92(3):e12924. doi: 10.1111/sji.12924 (PMC7507180; doi:10.1111/sji.12924)
Supplement: Supplementary file 1 — Appendix S1 [file SJI-92-e12924-s001.docx]

**Long-term *in-vitro* persistence of magnetic properties after magnetic bead-based cell separation of T cells**

Aicha Laghmouchi^1^, Conny Hoogstraten^1^, Peter van Balen^1^, J.H.Frederik Falkenburg^1^ and Inge Jedema^1^

***Supporting Information***

**Methods**

*Isolation procedure using CD45RA-Fab Streptamers*

CD45RA-Fab Streptamers were generated by the incubation of Strep-tagged CD45RA Fab fragments (6-8000-208, IBA Lifesciences, Göttingen, Germany) with Strep-Tactin magnetic microbeads (6-8000-208, IBA Lifesciences) in IS buffer; phosphate-buffered saline (PBS; 3623140, B. Braun, Melsungen, Germany) supplemented with 0.4% human serum albumin (HSA; H 163 NED, Sanquin Reagents, Amsterdam, The Netherlands), for 45 minutes at 4 ˚C on a MACSmix™ tube rotator (Miltenyi Biotec, Bergisch Gladbach, Germany). PBMC (Supporting Figure 8A) were washed with 4 ˚C IS buffer, and mixed with the CD45-Fab Streptamers and incubated for 20 minutes at 4 ˚C on the tube rotator. The tube with the mix of cells and Streptamers was applied onto the StrepMan Magnet (IBA) for 3 minutes. The supernatant with the unbound cells was removed to a new tube. New IS buffer was added to the bound cells and again placed onto the StrepMan Magnet for 3 minutes. The supernatant was again removed to a new tube and these steps were repeated another time, all the supernatants with unbound cells were pooled for counting and flow cytometry analysis (Supporting Figure 8B). The tube with the bound cells was taken from the magnet and new IS buffer was added to perform to dissociation step; removing the Strep-Tactin microbeads from the cells, using 1mM D-Biotin (6-8000-208, IBA). After incubation of 10 minutes at 4 ˚C, the tube was placed on the StrepMan Magnet for 3 minutes. The supernatant was removed to a new tube which should contain the dissociated CD45RA^pos^ cells. The cell suspension was resuspended in 0.5mM D-Biotin in PBS and incubated for 10 minutes at 4 ˚C and placed on the StrepMan Magnet for 3 minutes. The supernatant of this step was pooled with the previously obtained CD45RA^pos^ cells (Supporting Figure 8C). To remove the Fab fragments from the cells, cells were resuspended in 4 ˚C IS buffer an incubated for 10 minutes at 4 ˚C so the fragments could dissociate spontaneously. To check the removal of the Streptamers, the CD45RA^pos^ cells were again placed on the StrepMan Magnet and the fractions were then counted and measured. The gating procedure was performed after applying fitting instrument settings and compensation. The gating strategy of the different cell fractions started with the gating of lymphocytes using forward and sideward scatter and was followed by plotting for CD45RO (CD45RO-FITC; MHCD45RO01, Clone UCHL1, Invitrogen, Grand Island, NY, USA) and CD45RA (CD45RA-APC; 550855, Clone HI100, BD Pharmingen, San Diego, CA, USA) expression.

**Results**

PB -MNC

Pan T cell isolation

T cells

CD45RO isolation

CD45RO^+^

Memory T cells

CD45RA depletion

CD45RA^-^

Memory T cells

Day 0: PKH labeling

Day 14: Apply on MACS columns, without additional magnetic labeling

No

proliferation

Partial

proliferation

Full

proliferation

Day 14: FACS labeling pre- and post-application on MACS columns

Day 0: PKH labeling

Day 7: Cell surface FACS labeling for MACS beads

Day 0: co-culture T cells with HLA-mismatched EBV-LCL

Day 13: re-stimulation T cells with HLA-mismatched EBV-LCL

Day 14: CD137 magnetic bead labeling and MACS separation

**B**

**C**

**D**

**A**

Full

proliferation

Partial

proliferation

No

proliferation

Supporting Figure 1. Schematic overview of the methods as described in the Materials and Methods section.

**
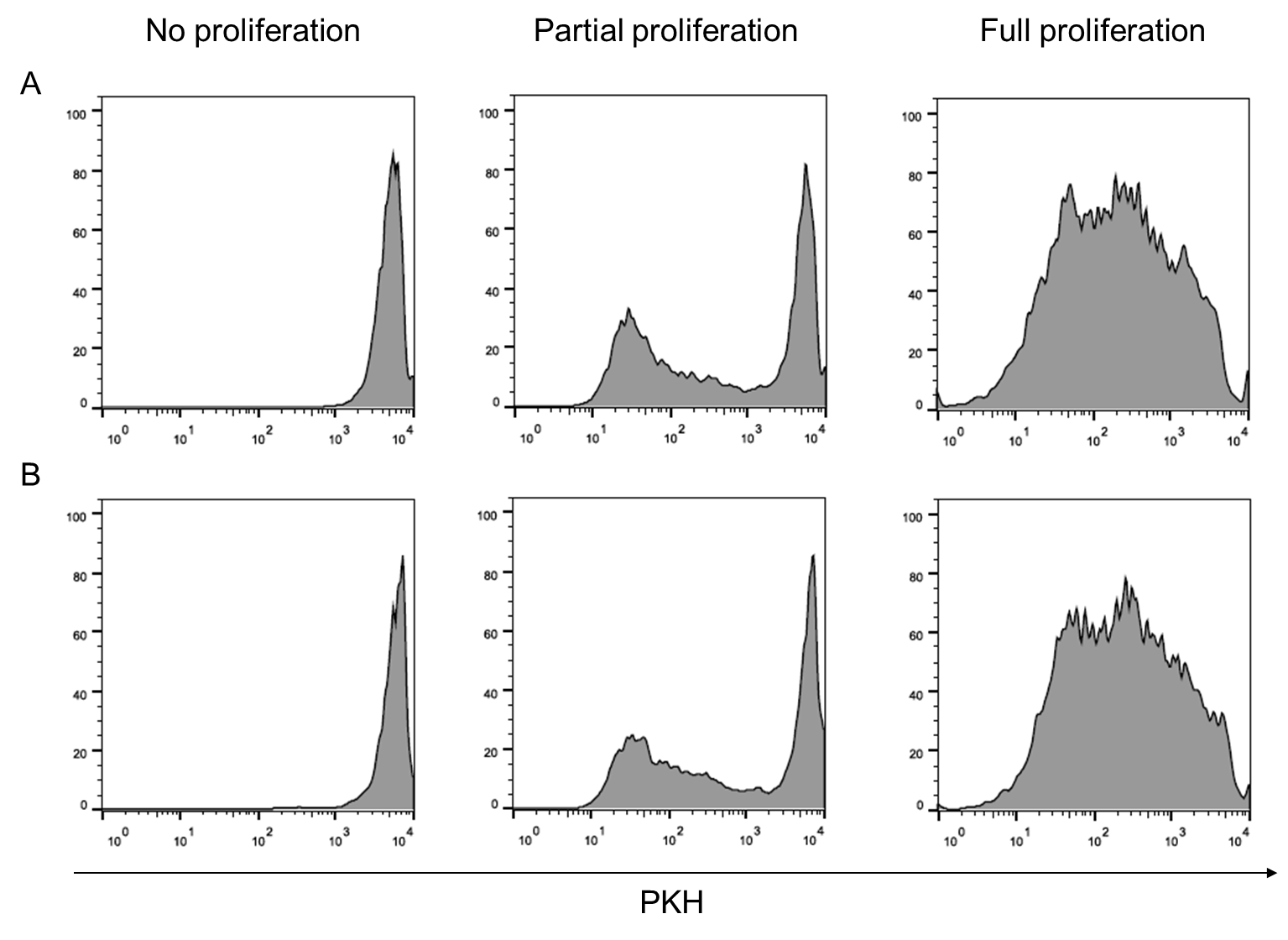
**

Supporting Figure 2. Representative histograms showing the proliferation of A. positively selected and B. untouched isolated memory T cells after two weeks of *in-vitro* culture under different stimulation conditions (from left to right: no/minimal proliferation (cytokines only); proliferation of a portion of T cells (allo-reactive T cell response), and full, a-specific proliferation of almost all T cells (PHA stimulation)). Lymphocytes were gated using the forward and sideward scatter after which CD3^+^ cells were selected to be plotted as histograms to analyze the PKH labeling, with the median fluorescence intensities (MFI) of PKH indicated in the histograms.

MFI: 197

MFI: 234

MFI: 795

MFI: 793

MFI: 5738

MFI: 5037


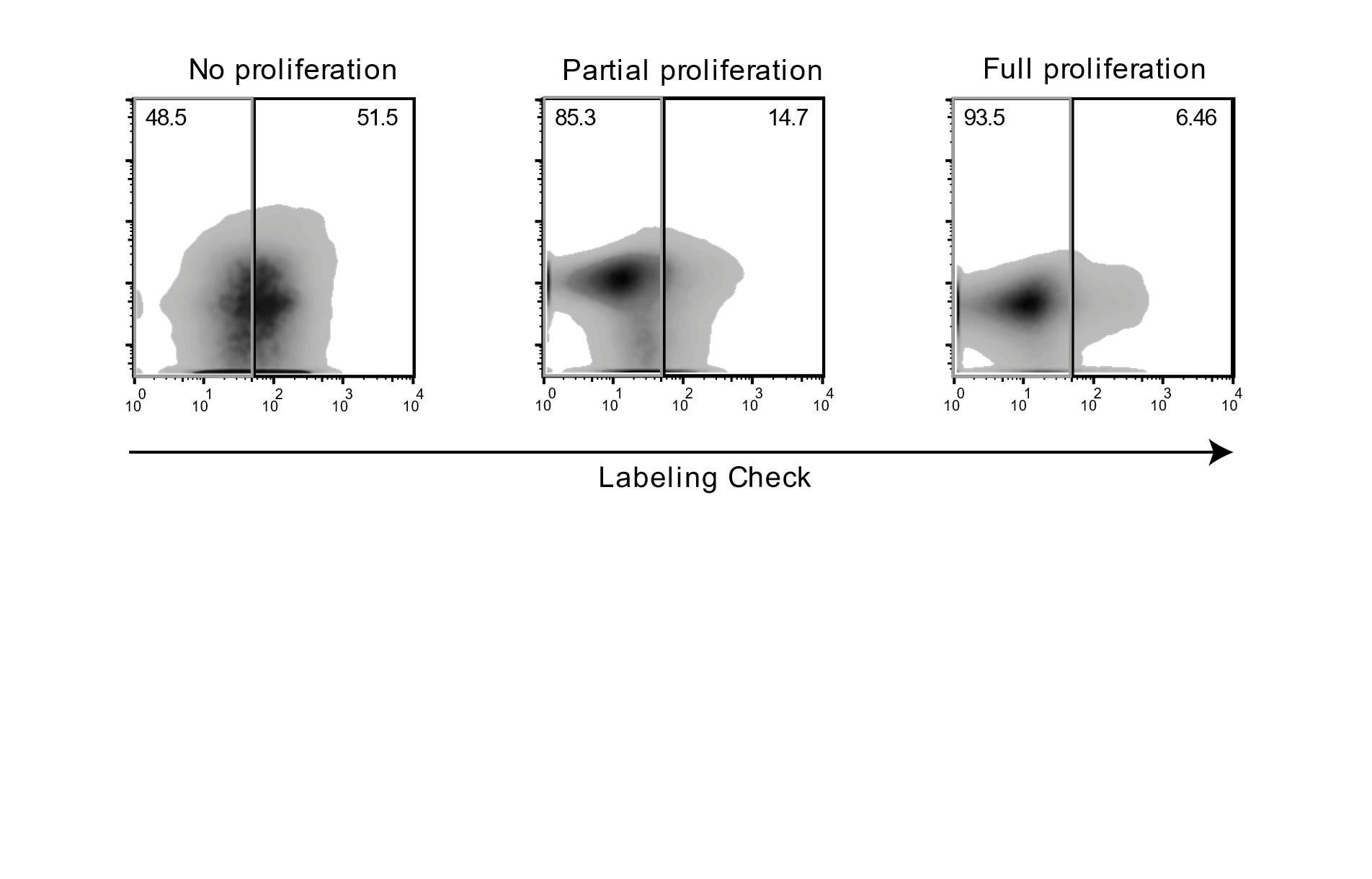


Supporting Figure 3. Positively selected memory T cells retain magnetic nanoparticles on their cell surface. Representative density plots of viable cells show the Labeling Check reagent staining (positive for staining indicated by the black boxes and negative by the grey boxes) after one week of *in-vitro* culture under different stimulation conditions (from left to right: no/minimal proliferation (cytokines only); proliferation of a portion of T cells (allo-reactive T cell response), and full, a-specific proliferation of almost all T cells (PHA stimulation)). The gating of lymphocytes was based on forward and sideward scatter after which the CD3^+^ cells were selected and plotted for Labeling Check staining.

**
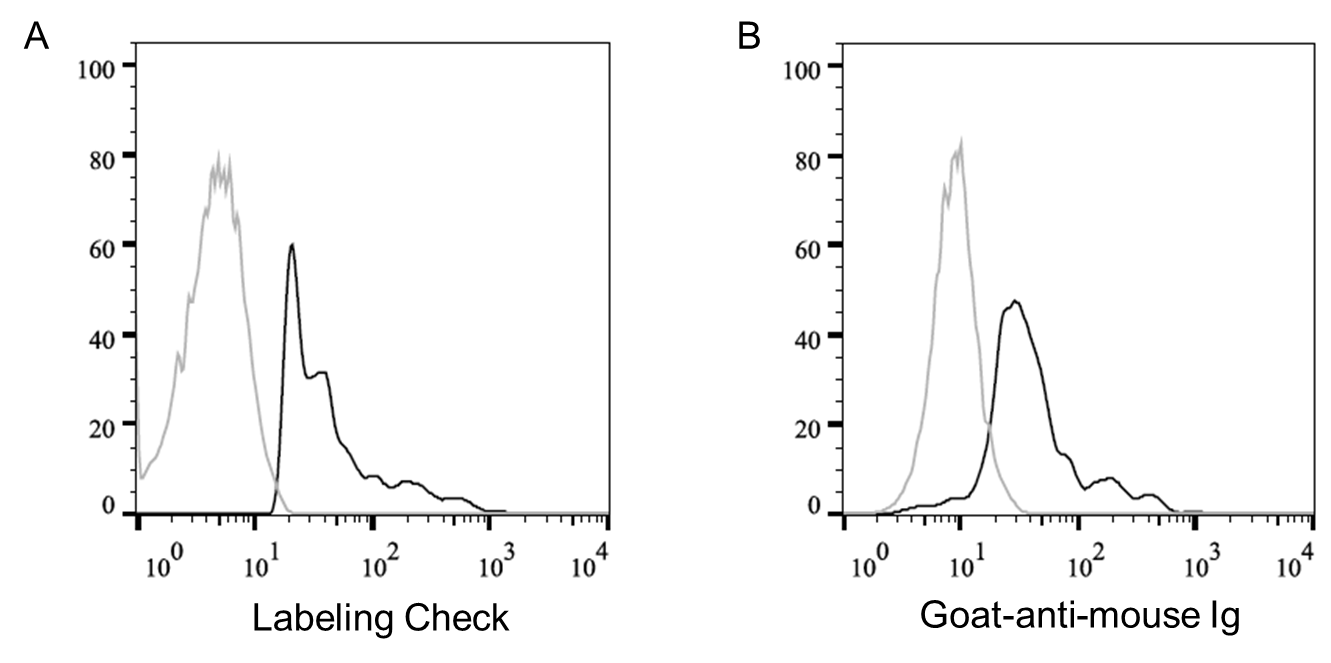
**

Goat-anti-Mouse Ig

Labeling Check

Supporting Figure 4. Cells with persistent magnetic nanoparticles (Labeling Check positive) also show presence of monoclonal antibodies on cell surface after two weeks of cell culture. Representative histograms (of n=3) are shown in which the magnetically isolated Labeling Check positive memory T cells (black curves) and Labeling Check negative memory T cells (grey curves) are plotted for the staining with A. Labeling Check reagent that stains specifically the magnetic nanoparticles and with B. goat-anti-mouse-Ig antibodies that stain the monoclonal antibodies by which the magnetic nanoparticles bind to the cell surface. Lymphocytes were gated using the forward and sideward scatter followed by selection of CD3^+^ cells to generate the overlay of histograms.


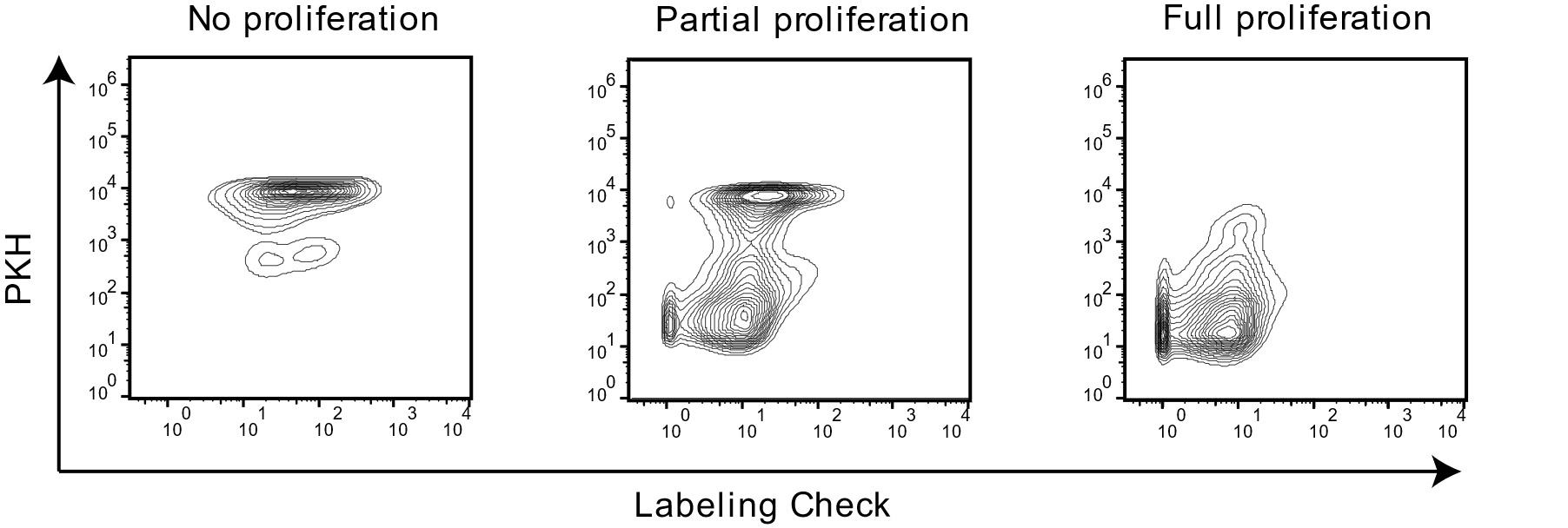


Supporting Figure 5. Non-divided, positively selected memory T cells retain magnetic nanoparticles on their cell surface. To investigate the correlation between proliferation and the staining with Labeling Check, contour plots were made of PKH staining against Labeling Check staining (representative of n=3 is shown). From left to right: no/minimal proliferation (cytokines only); proliferation of a portion of T cells (allo-reactive T cell response), and full, a-specific proliferation of almost all T cells (PHA stimulation)). The gating of lymphocytes was based on forward and sideward scatter and cells were then plotted for PKH and Labeling Check staining.

Supporting Figure 6. Non-divided, positively selected memory T cells retain magnetic nanoparticles also intracellularly. From a representative experiment (n=3), after two weeks of culture, positively selected memory T cells were analyzed for the presence of magnetic particles using Labeling Check reagent. Surface labeling with Labeling Check reagent (grey curve) was compared to the surface labeling of untouched isolated memory T cells (grey dashed curve). When additional intracellular labeling was performed, the positively selected memory T cells showed an even higher staining intensity (black curve). The gating was first done on lymphocytes using the forward and sideward scatter followed by the selection of CD3^+^ cells to generate histograms and to compare the Labeling Check staining of the different T-cell populations an overlay was generated. The median fluorescence intensities (MFI) of the Labeling Check staining are indicated .


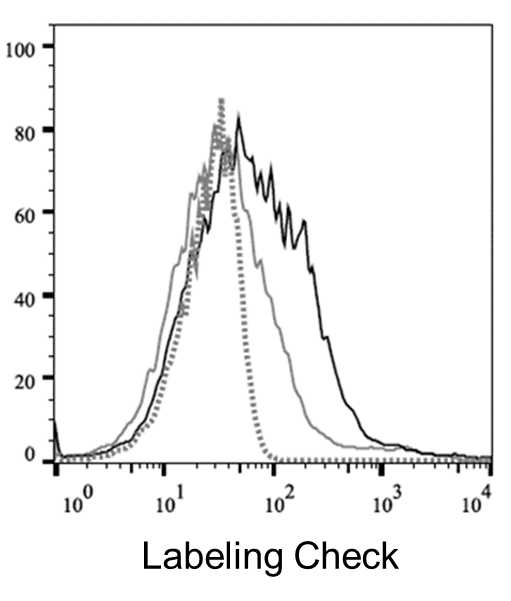


27.8

34.1

59.4


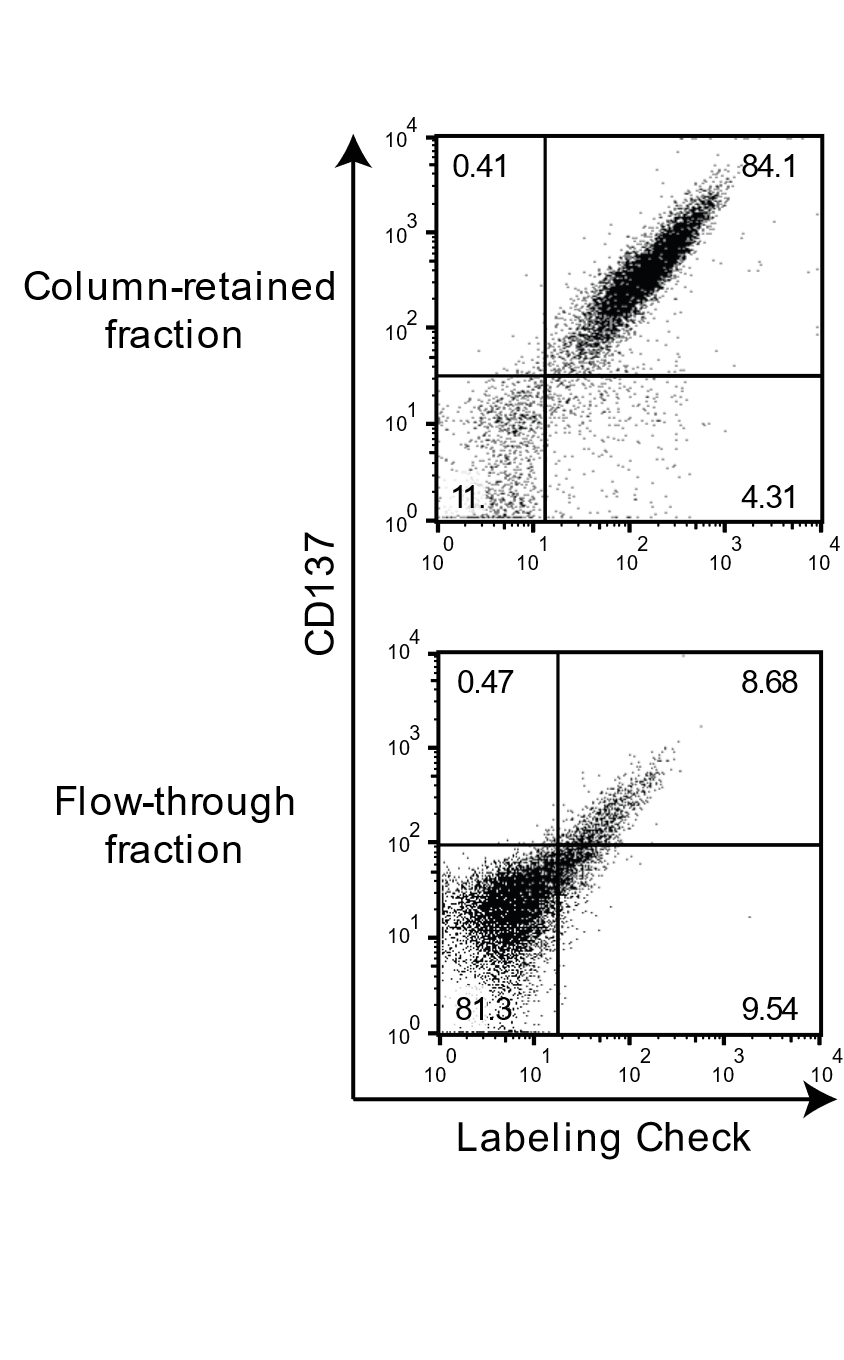


Supporting Figure 7. CD137^pos^ isolated T cell populations are Labeling Check reagent positive. Two weeks after primary stimulation memory T cells were restimulated with HLA-mismatched EBV-LCL to perform a CD137 MACS isolation the next day. Dotplots are shown of a representative example (n=3) of the untouched isolated memory T cells, the upper dot plot shows that the CD137^pos^ (column-retained) fraction is double positive for CD137 and Labeling Check reagent and the lower dot plot, the CD137^neg^ (flow-through) fraction does not stain for CD137 and not for Labeling Check reagent. The fractions were analyzed by gating the lymphocytes using forward and sideward scatter. The CD3^+^ cells were selected and plotted for CD137-APC and Labeling Check reagent-PE staining.


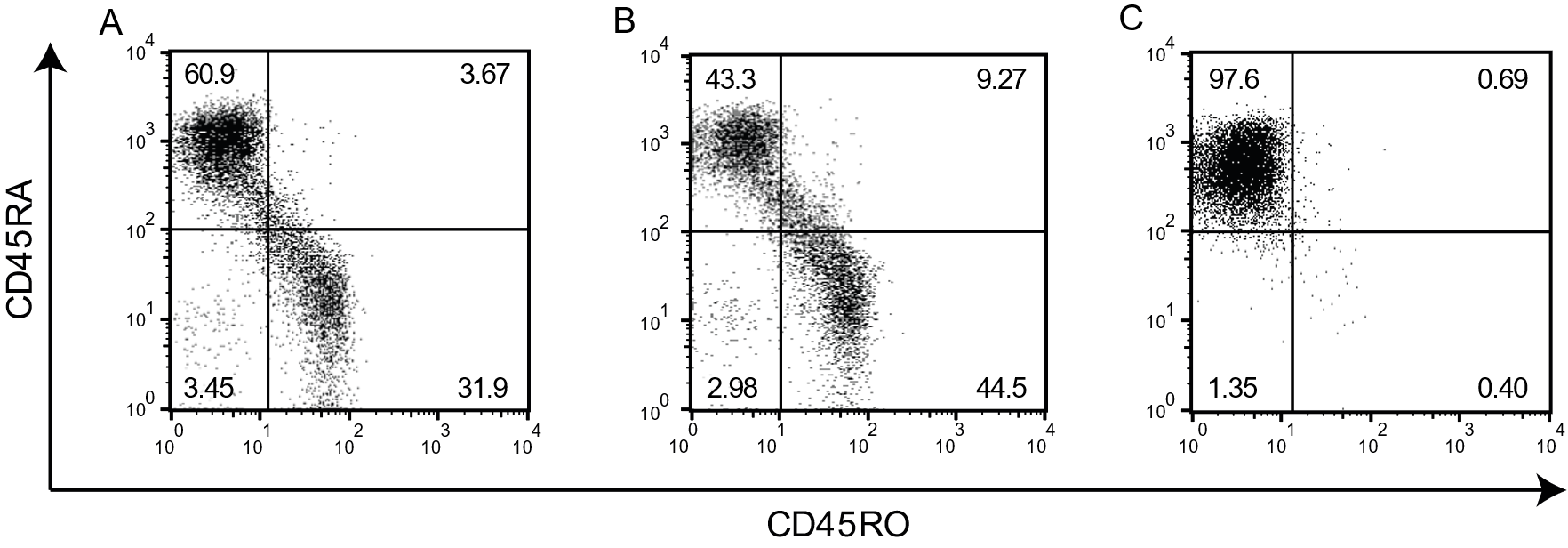


Supporting Figure 8. Reversible magnetic isolation using CD45RA-Fab Streptamers. A. Dot plot of PBMC when labeled with antibodies against CD45RA and CD45RO. These PBMC were incubated with CD45RA-Fab Streptamers and applied to the StrepMan Magnet. B. The supernatant consisted of unbound cells, when labeled with CD45RA and CD45RO antibodies showing unbound CD45RA^pos^ cells (decreased compared to starting population) and CD45RA^neg^ cells. C. The cells with the CD45RA-Fab Streptamers bound to the magnet, and after the dissociation step to remove the magnetic microbeads, all CD45RA^pos^ cells were magnetic-microbeads-free. This was confirmed by applying the cells against the magnet and no cells could be counted or measured in the magnet-bound fraction. The different cell fractions were analyzed by gating on lymphocytes using the forward and sideward scatter and the cells were then plotted for CD45RO and CD45RA expression.
